# Effect of Calstabin1 Depletion on Calcium Transients and Energy Utilization in Muscle Fibers and Treatment Opportunities with RyR1 Stabilizers

**DOI:** 10.1371/journal.pone.0081277

**Published:** 2013-11-26

**Authors:** Anke Breckner, Magdalena Ganz, David Marcellin, Jens Richter, Nicole Gerwin, Martin Rausch

**Affiliations:** 1 Global Imaging Group, Novartis Institutes for BioMedical Research, Basel, Switzerland; 2 Bioimaging Center, University of Konstanz, Konstanz, Germany; 3 Musculoskeletal Disease Area, Novartis Institutes for BioMedical Research, Basel, Switzerland; Baylor College of Medicine, United States of America

## Abstract

Depletion of calstabin1 (FKBP12) from the RyR1 channel and consequential calcium leakage from the sarcoplasmic reticulum (SR) is found in certain disease conditions such as dystrophy, aging or muscle overuse. Here, we first assessed the effect of calstabin1 depletion on resting Ca^2+^ levels and transients. We found that depletion of calstabin1 with the calstabin1-dissociation compound FK506 increased the release of calcium from the SR by 14 % during tetanic stimulation (50 Hz, 300 ms) and delayed cytosolic calcium removal. However, we did not find a significant increase in resting cytosolic Ca^2+^ levels. Therefore, we tested if increased SERCA activity could counterbalance calcium leakage. By measuring the energy utilization of muscle fibers with and without FK506 treatment, we observed that FK506-treatment increased oxygen consumption by 125% compared to baseline levels. Finally, we found that pretreatment of muscle fibers with the RyR1 stabilizer JTV-519 led to an almost complete normalization of calcium flux dynamics and energy utilization. We conclude that cytosolic calcium levels are mostly preserved in conditions with leaky RyR1 channels due to increased SERCA activity. Therefore, we suggest that RyR1 leakiness might lead to chronic metabolic stress, followed by cellular damage, and RyR1 stabilizers could potentially protect diseased muscle tissue.

## Introduction

Fast calcium release and reuptake during muscle activation is tightly regulated to ensure functional excitation contraction coupling (ECC). Calcium release from the SR into the cytoplasm is controlled by the RyR1 receptor. After muscle fiber contraction, cytosolic free calcium is typically removed quickly by a) SERCA, pumping calcium back into SR, and b) calcium binding proteins, such as parvalbumin in fast twitch muscle fibers [1].

Leakiness of the RyR1 channel is assumed to cause dysfunction of muscle fibers and might finally lead to cellular damage and cell death. One cause for leakiness of the RyR1 channel is depletion of calstabin1 from the RyR1 channel [2]. This link has been studied in cellular systems by measurements of the open-probability of the RyR1 receptor before and after chemical depletion of calstabin1 using FK506 or rapamycin [3][4]. In animal models, depletion of calstabin1 has also been observed for several disease conditions including myocardial infarction [5], muscular dystrophy [6], aging [7] and muscle overuse [8].

In the chronic disease conditions, reduced maximal force was observed in parallel with RyR1 leakiness. However, when isolated muscle was preincubated with rapamycin, an acute increase in caffeine-induced tetanic force was observed [3]. Hence, in contrast to chronic RyR1 leakiness, acute RyR1 leakage does not seem to lead to failure of calcium-activated force.

Therefore, an open question remains how RyR1 leakiness affects calcium release from the SR and cytosolic calcium levels under resting conditions. As stated by the “cell boundary theorem”, in steady state conditions intracellular alterations, such as RyR1 leakiness, cannot change the cytosolic resting calcium concentration [9]. Thus, RyR1 leakage might lead to not only a change of the calcium release but also reuptake dynamics during muscle fiber activation and that calcium leakage from the SR under resting conditions is counterbalanced by increased SERCA activity. 

We studied if these physiological alterations could be reversed by treatment with compounds that are known to stabilize the closed state of the RyR1 receptor. The benzothiazepine derivative JTV-519, also known as K201, is a compound with known RyR1 stabilizing properties [10,11]. In mice with myocardial infarction treatment with JTV-519 enhanced RyR1‑calstabin1 binding, restored skeletal muscle RyR1 channel function and decreased muscle fatigue [10]. Therefore, we tested how treatment with JTV-519 affects calcium dynamics after calstabin1 depletion by FK506 [12].

To address these questions, we measured calcium resting levels and calcium release during muscle fiber activation using 2-photon line scan imaging. In addition, we determined the oxygen consumption rate as an indicator for potential changes in SERCA activity using an extracellular flux analyzer.

## Materials and Methods

### Ethics statement

All animal work was conducted according to national and international guidelines and approved by the cantonal veterinary services Basel Stadt.

### Fiber preparation

The flexor digitorum brevis (FDB) muscle was dissected manually from adult 6-9 weeks old male C57BL/6 mice, which were killed by decapitation after anaesthetizing them with isoflurane (4% in air). The FDB muscle was enzymatically dissociated for one hour in Tyrod’s buffer (138 mM NaCl, 2 mM CaCl_2_, 1mM Mg Acetate, 4 mM KCl, 5 mM Glucose, 10 mM HEPES, pH 7.4) containing 2.2 mg/ml collagenase I (Sigma) in the incubator at 37 °C and 5% CO_2_. After incubation with collagenase, muscle fibers were manually isolated using fire polished pipette tips and transferred onto laminin coated cover slips. The muscle fibers were kept in Dulbecco’s Modified Eagle Medium (DMEM) supplemented with 10 % FCS and 1 % Penicillin-Streptomycin in the incubator at 37°C for 3‑4 hours before medium was exchanged. For JTV-519 pretreatment, the fresh medium was supplemented with the respective concentration of the compound and the fibers were kept in the incubator for 12 hours before starting measurements. 

### Staining of FDB fibers with calcium indicator mag-fluo-4 to measure Ca^2+^ transients

FDB fibers were stained with mag-fluo-4 AM (K_D_~22 µM, Invitrogen) dissolved in Tyrod’s buffer for 20 min at room temperature (20.4 °C). The final concentration of the dye in buffer was 5 µM. During the calcium measurements N-benzyl-p- toluene sulphonamide (BTS, Sigma) was added to the buffer at a concentration of 10 µM to prevent muscle fiber contractions, without affecting calcium release. 

### Staining of FDB fibers with calcium indicator fura-2 to measure baseline Ca^2+^ levels

FDB fibers were stained with fura-2 AM (K_D_~0.26 µM, Invitrogen) dissolved in Tyrod’s buffer for 60 min in the incubator (37°C, 5% CO_2_). The final concentration of the dye in buffer was 5 µM.

### Fluorescence imaging for the analysis to measure Ca^2+^ transients

For evaluation of the calcium signals an upright Olympus FV1000-BX61 microscope equipped with a MaiTai HP DeepSee 2-photon laser was used in combination with a XLPLN 25x/NA1.05 water immersed IR corrected lens. The excitation wavelength was 970 nm. Emission was detected on two channels: A 480-490 nm filter was used to detect the second harmonic signal (SHG), which was used to detect potential fiber contractions [13][14]. Fluorescence of the calcium dye was detected at 515-560 nm. Bi-directional line scan mode with a temporal resolution of 0.244ms/line was used to measure the temporal profile of the fluorescence signal. All experiments were carried out at room temperature, which was kept constant at 20.4 °C. FDB fibers were stimulated with 0.2ms electrical pulses. We used either single pulses for isolated twitches or pulse trains (300ms at 50Hz) for tetanic stimulation. For single twitch measurements we recorded 4000 lines, for pulse train experiments 8000 lines were recorded. The minimum time between two consecutive stimulations was 2 min. In the first half of the experiment (up to 30 min) the calcium signal was measured under control conditions. After that calstabin1 was depleted by incubating muscle fibers with FK506 for 20 min at a dose of 25 µM as published previously [3,15,16]. After an incubation of 20 min the next series of muscle fibers was studied (up to 20 min). Each muscle fiber was just measured one single time. In addition to the standard design (control-treatment) a few experiments with burst activation were carried out with the design (control-control) to exclude potential time effects (e.g. loss of function with time; data not shown). 

### Fluorescence imaging for the analysis to measure resting Ca^2+^ levels

Resting Ca^2+^ levels were measured on a Zeiss Axiovert S100TV inverted microscope (Carl Zeiss, Jena, Germany) equipped with a NeoFluar 20x/NA 0.17 water immersed lens using wide field illumination at 340nm and 380nm. Fluorescence emission of the calcium indicator was detected at 500-530 nm using a Cascade 128+ CCD camera (Photometrics, Tucson, USA).

A calibration curve for the fura-2 signals was generated using a calibration kit from Molecular Probes (Catalogue N° F6774). In brief, baseline corrected ratio images were generated for eight different Ca^2+^ concentrations ranging from 0 to 351 nM. A linear regression analysis was performed on these eight data points to derive the parameters to calculate the Ca^2+^ concentration from ratio images.

### Quantitative and statistical analysis

The fractional change ΔF/F was calculated from the raw data. The decay phase of ΔF/F was parameterized using a non-linear least squares fit for a two-exponential decay model based on the Levenberg-Marquardt algorithm. All statistical tests were based on a mixed model analysis (SYSTAT 13). Comparisons were made for the amplitude, fast and slow decay parameter (ΔF/F data from single twitch experiments). For the tetanic stimulation, we analyzed the area-under-the curve (AUC). In each statistical analysis, the treatment was used as the fixed factor. Furthermore, the “muscle-ID” was included as a random factor to correct for multiple comparisons. This correction was necessary because several FDB fibers originated from the same animal. 

### Energy utilization in isolated muscle fibers

Energy utilization was measured using the Seahorse XF Extracellular Flux Analyzer for 24-well plates according to the Seahorse standard protocol (http://www.seahorsebio.com/resources/tech-writing/techbrief-intact-muscle-fiber.pdf). 

Muscle fiber preparation was performed as described. However, all fibers dissociated from four FDB muscles were pooled for equal distribution to all wells of the XF cell culture microplate. Fibers were treated for 12 hours over night by exchanging the culture medium by medium supplemented with JTV-519. The oxygen consumption rate was measured during 60mins. FK506 (25µM) was added after 20mins. Muscle fibers were not activated during the whole experiment.

## Results

### Delayed Ca^2+^ removal from the cytoplasm after FK506 treatment

**Figure 1:**
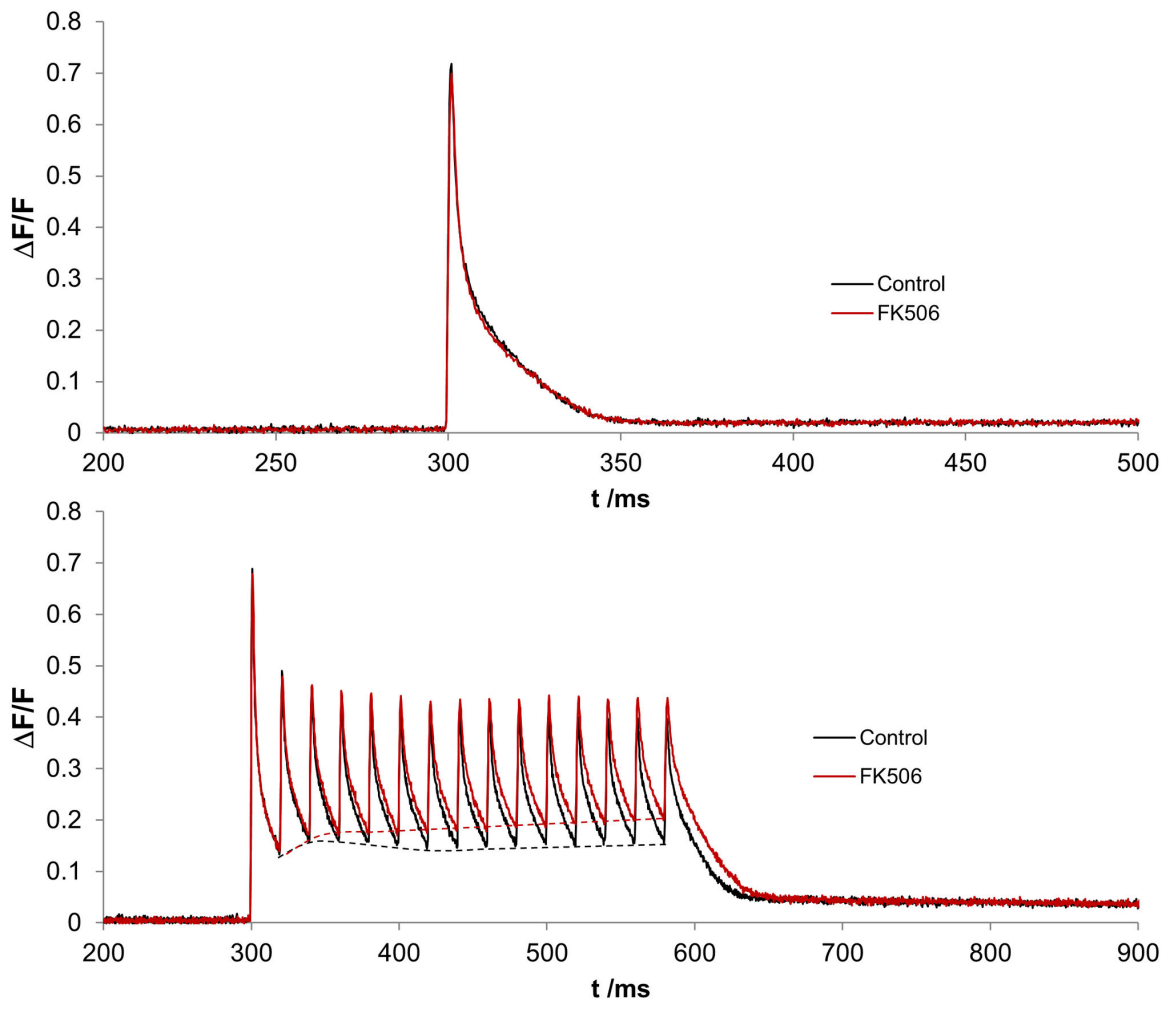
Temporal profile curves for calcium transients during single twitch activation (A) and tetanic stimulation (B). Each line corresponds to the average of all single fiber measurements that have been included in the statistical analysis. For improving the visibility of group differences, the time interval was adjusted accordingly. The red and black dashed lines indicate the drift of the baseline calcium during the tetanic stimulation. Treatment of fibers with 25 μM FK506 (red line) leads to an elevated inter-peak-baseline level compared to untreated control fibers (black line).

We first studied the effect of the calstabin1-dissociation compound FK506 on the calcium dynamics after single twitch stimulation (Figure 1A) and tetanic stimulation at 50 Hz (Figure 1B). We evaluated the signal of two groups: control (n=53) and FK506 (n=52) for single twitch activation; and control (n=42 fibers) and FK506-treated (n=42 fibers) for tetanic stimulation, each isolated from 8 FDB muscles in total. The differences in fiber number between single twitch and burst activation arose from detachment of some fibers during burst activation resulting in not-evaluable profiles.

The effect of FK506 (25µM) was minimal for single twitch activation (Figure 1A). We found no statistical significant difference in calcium release and uptake between untreated and FK506 treated fibers. However, we observed a difference in calcium kinetics during burst activation (Figure 1B). Here, a small delay of cytosolic calcium removal was measured, leading to a consecutive elevation of the inter-peak-baseline levels. Overall, the area under the curve (AUC) increased by 14 % (p<0.001) upon treatment with FK506 after tetanic stimulation. 

### Effect of RyR1 stabilizing compound JTV-519

We next studied the RyR1 stabilizer compound JTV-519 (ranging from 0.1 to 10 um) for its effect on calcium transients. We evaluated the signals from four groups of fibers: Control (n=42 fibers), FK506-treated (n=42 fibers), JTV-519-treated (n=53 fibers) and JTV-519 + FK506-treated (n= 44 fibers), isolated from 8 FDB muscles in total.

We found that pretreatment of fibers with 1μM JTV-519 for 12 hours led to a normalization of the calcium flux dynamics upon FK506 treatment, suggesting a RyR1 stabilizing effect of JTV-519. Lower concentrations of JTV-519 were found to be ineffective, whereas higher concentrations led to a partial or even complete inhibition of calcium release (data not shown).

**Figure 2:**
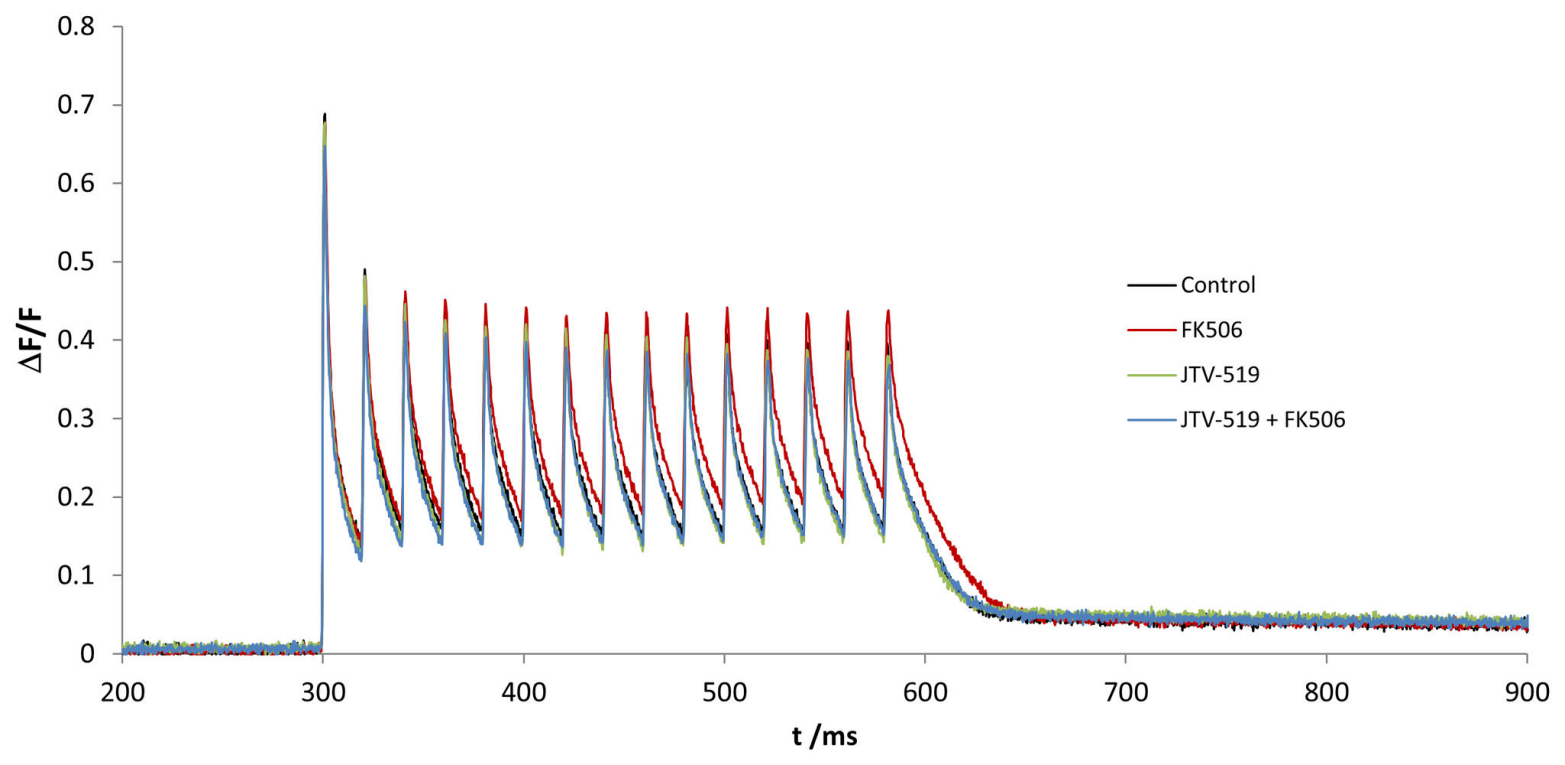
Averaged calcium profiles for burst activation. Each line corresponds to the average of all single fiber measurements that have been included in the statistical analysis. For improving the visibility of group differences, the time interval was adjusted accordingly. The effect of FK506 on JTV-519 pretreated compared to non-pretreated fibers is shown. Pretreatment with 1 μM JTV-519 leads to normalization of the calcium flux to control levels after FK506 treatment.

**Figure 3:**
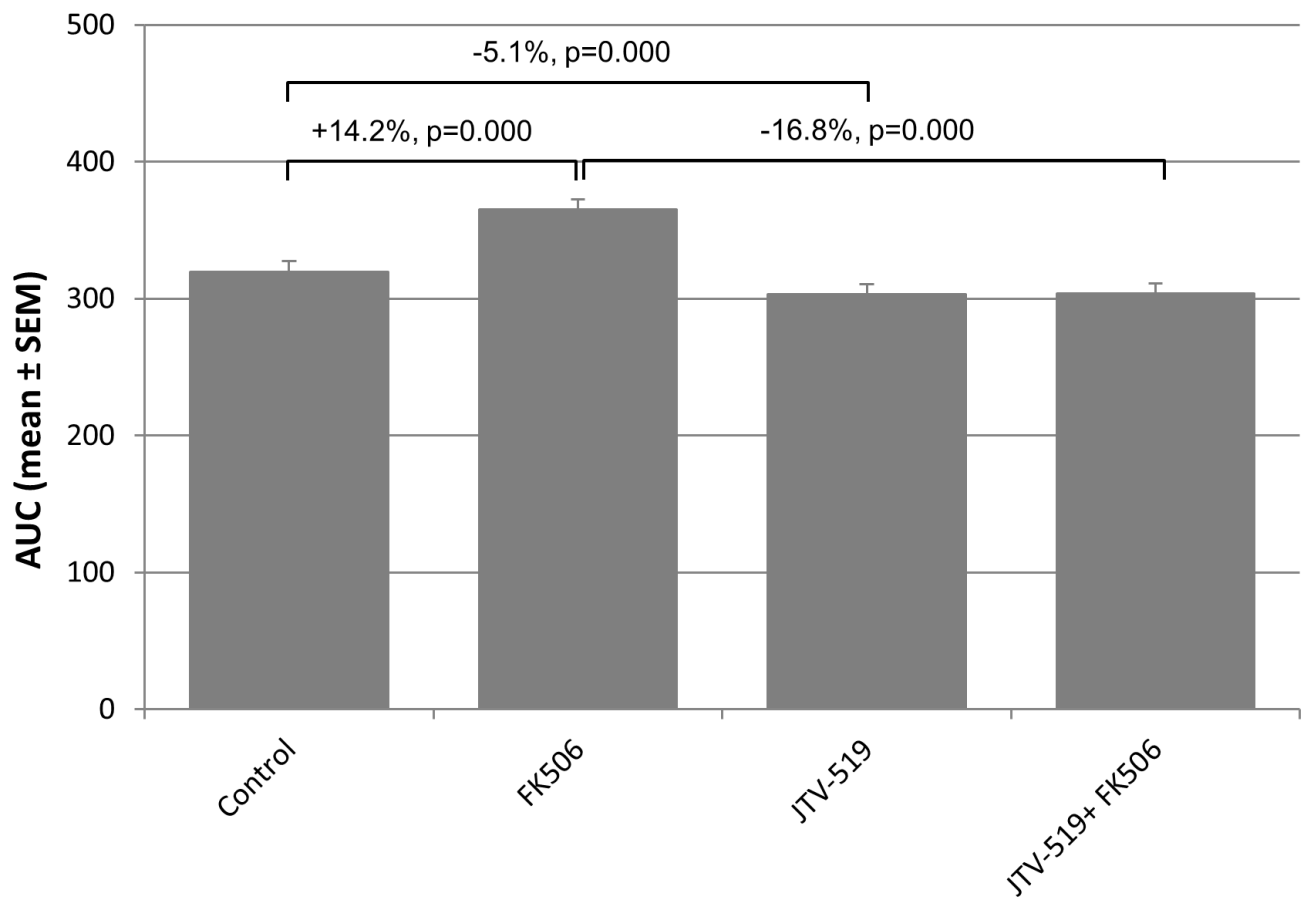
The graph shows the estimates for the AUC (mean±SEM) for the four groups. Changes, displayed in %, are statistically valuated by the corresponding p-value. n: number of fibers measured per group. Control (n=42), FK506 (n=42), JTV-519 (n=53) and JTV-519 + FK506 (n= 44).

In non-pretreated fibers FK506 treatment led to delayed of calcium reuptake (Figure 2, red line) whereas JTV‑519 pretreatment inhibited the FK506 effect (Figure 2, blue line). Overall, we found a significantly reduced AUC in JTV-519 pretreated fibers (-17 %, p<0.001) after FK506 treatment compared to non-pretreated fibers (Figure 3). We also observed a small but significant reduction of the AUC (-5 %, p<0.050) in JTV‑519 pretreated fibers compared to control fibers.

#### Cytosolic resting Ca^2+^ levels

We analyzed the potential effects of FK506 on cytosolic resting Ca^2+^ levels using the calcium indicator fura-2. The resting calcium concentration was measured after keeping the fibers for 20min in Tyrod’s buffer (control group) or 25 µM FK506 in Tyrod’s buffer (FK506 treatment) at room temperature. 40 fibers from 4 different animals were analyzed for each group. The calcium concentration was 73.5nm±16.3 (mean±stdev) for the control group and 67.9nm±19.5 (mean±stdev) for FK506 treatment. There was no statistical significant difference between the groups (p=0.188). 

### Increased energy utilization induced by FK506 treatment is partially inhibited by JTV-519

**Figure 4:**
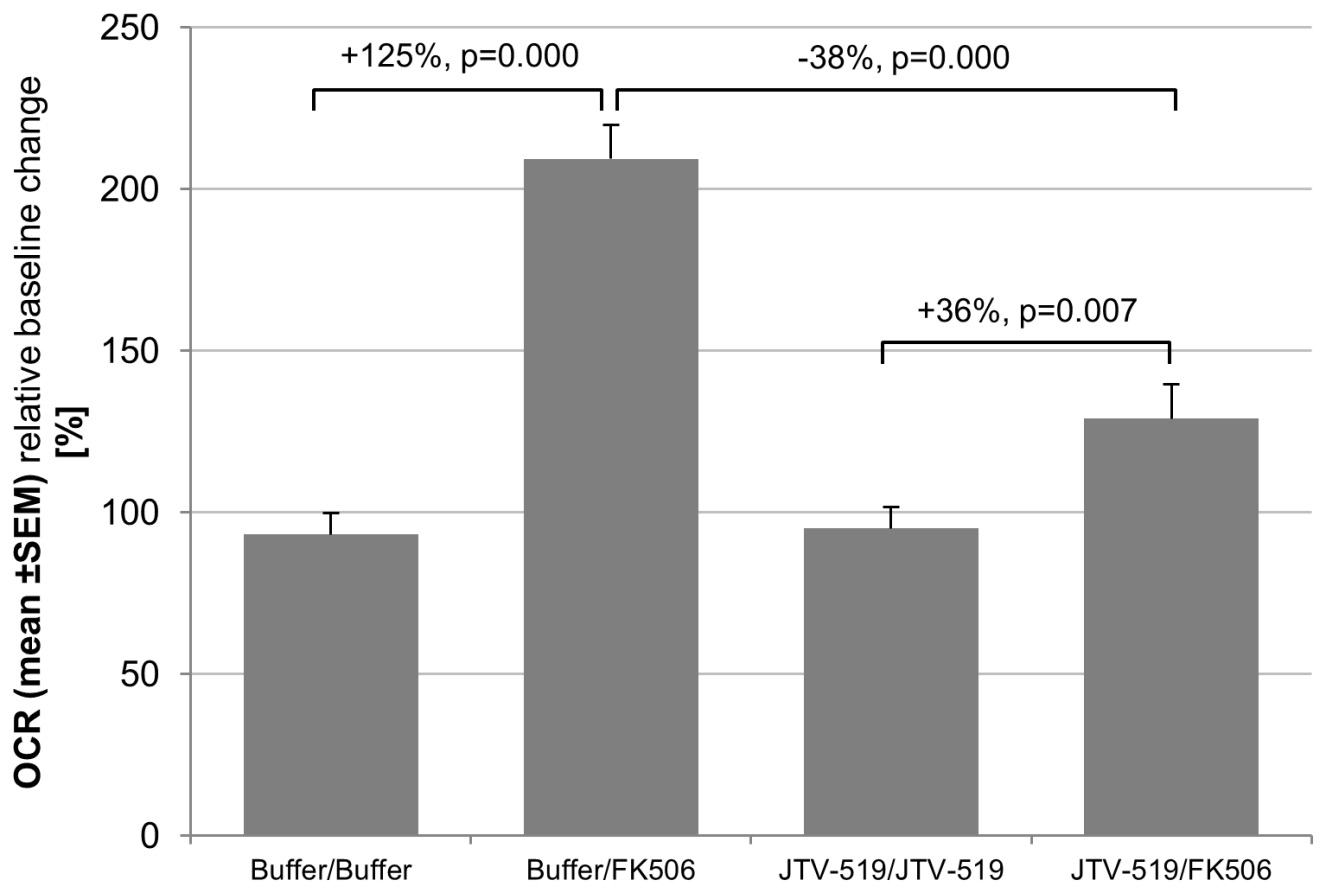
Energy utilization in FDB fibers. The graph shows the estimates for the relative baseline change ± SEM after different treatment conditions (pretreatment/injection). Changes, displayed in %, are statistically valuated by the corresponding p-value. The increased SERCA activity observed after FK506 injection can be incompletely normalized by pretreatment with JTV-519.

To address the potential enhancement of SERCA activity, we measured the energy utilization under resting conditions of the muscle fibers before and after calstabin1 dissociation by FK506 (Figure 4). The oxygen consumption rate (OCR) was measured for about 20 min to obtain baseline levels before the treatment compound was injected and the OCR was measured for another 20 min. The treatment effect was calculated as relative baseline change. 

We found a significant elevation of the OCR (125 %, p<0.001) upon FK506 treatment. However, pretreatment with the RyR1 stabilizer JTV-519 overnight led to a significant reduction of the OCR (-38 %, p<0.001) upon FK506 treatment compared to non-pretreated fibers. As JTV-519 treatment alone led to no significant change compared to control (p=0.845), unspecific effects of the compound on the energy utilization can be excluded. 

## Discussion

It has been suggested that calstabin1 dissociation from the RyR1 leads to an increased open probability and increased calcium leakage from the SR into the cytoplasm. This event has been described in mouse models for certain muscle conditions including myocardial infarction [5], Duchenne muscular dystrophy [6], aging [16] and muscle overuse [8]. As previously published, we used the immunosuppressive drug FK506 to induce calstabin1 dissociation from RyR1 and study the effects of RyR1 leakiness [3,17]. An increased open probability of the RYR1 receptor could have different mechanistic effects for the calcium handling during rest and activation of FDB fibers: 1) it could lead to an elevation of cytosolic resting Ca^2+^ levels or 2) it might change Ca^2+^ release during activation. In this study and in line with the cell boundary theorem, we found no significant increase in cytosolic resting Ca^2+^ levels. 

It is important to note that accurate measurement of the temporal profiles of electrically evoked calcium transients is key for detecting the subtle differences in calcium reuptake rates. For measuring the calcium dynamics, we used mag-fluo-4 calcium dye. As the temporal change in cytosolic calcium concentration (Δ[Ca^2+^]) is in the millisecond range [18], only a low-affinity indicator can accurately determine the Δ[Ca^2+^] temporal profile. Furthermore, in contrast to high-affinity dyes, low-affinity dyes are not buffering intracellular calcium, do not perturb the calcium transients [19] and show a linear relationship between calcium binding and fluorescence for calcium concentrations significantly above 1µM. Hollingworth et al. assessed different low affinity calcium dyes for their ability to precisely track the kinetics of Δ[Ca^2+^] in skeletal muscle [20]. Mag-fluo-4 was found to be the only low-affinity calcium dye with the ability to track Δ[Ca^2+^] with little or no kinetic delay and a signal/noise ratio of ΔF that is an order of magnitude larger than for the best studied dye Mag-fura-2 (furaptra), with visible excitation wavelengths and similar concentrations [20]. Finally, mag-fluo-4 proved to be optimal for two-photon excitation at 970 nm.

While calstabin1 depletion did not lead to a permanent elevation of cytosolic resting Ca^2+^ levels, the dynamic studies showed that the rate of calcium removal from the cytoplasm after repeated activation was slightly reduced compared the control FDB fibers. This was reflected by a continuous increase of the inter-peak cytosolic calcium levels and a significantly increased AUC. Persistent RYR1 leakage caused by calstabin1 removal with FK506 led to a permanent leakage of calcium from the SR into the cytoplasm. Under resting conditions, the persistent calcium efflux might be counterbalanced by increased SERCA activity and thereby preserve the normal resting Ca^2+^ levels in the cytoplasm. However, after repeated activation of the muscle fiber increased SERCA activity may not be sufficient to counterbalance RyR1 leakage and small differences would accumulate to become visible as a continuous increase of the inter-peak cytosolic calcium concentration. 

We tested this hypothesis by measuring the oxygen consumption rate using an extracellular flux analyzer and found an increase in energy utilization after FK506 treatment, suggesting that SERCA activity may be increased. This increase would be in line with observations by Gilchrist and colleagues for SR vesicles from rabbit fast twitch muscle [21]. They found a doubling of SERCA1 catalytic activity upon activation of the RyR1 receptor by ryanodine or ionomycin. Their data indicate that SERCA1 activity is highly sensitive to RyR1 leakiness and potently counterbalances cytosolic calcium release by RyR1. Moreover, the close proximity of terminal cisternae and mitochondria might lead to enhanced mitochondrial calcium uptake through the mitochondrial calcium uniporter (MCU). This would be followed by a direct stimulation of oxidative-phosphorylation and ATP production [22]. 

A similar observation was made by Durham and colleagues for myotubes derived from mice with a malignant hyperthermia mutation (Y552S). They found that resting cytosolic Ca^2+^ levels of the wild-type and mutant RYR1 myotubes were not significantly different at room temperature [23]. According to their interpretation, this might reflect the ability of SERCA to resequester local increases of Ca^2+^ due to leak from the RYR1. Our results support such a coupling of RyR1 and SERCA1 activity, but suggest also that in situations of tetanic stimulation SERCA activity is not sufficiently increased to counterbalance calcium leakage. 

Chronic calstabin1 depletion has been observed in several forms of muscle disease. It can therefore be assumed that a persistent increase of energy consumption and SERCA catalytic activity should also occur in these conditions. As a consequence, mitochondrial respiration and production of ROS could be elevated leading to oxidation of the RyR1 protein and damage to the RyR1 receptor function [16]. Further, general damage to cellular proteins by ROS can accelerate muscle ageing and loss of muscle function [24].

Restoration of RyR1-calstabin1 interaction and thereby reduction of SR calcium leakage might be a promising approach for preservation of muscle function. The 1,4-benzothiazepine derivative, JTV-519, discovered by Kaneko et al. was found to prevent dissociation of calstabin2 from RyR2, the cardiac isoform, and thereby inhibit spontaneous calcium leak in heart failure [25], [26]. Furthermore, Wehrens et al. demonstrated that enhanced RyR1-calstabin1 binding, restored skeletal muscle RyR1 channel function and decreased muscle fatigue in mice with myocardial infarction after treatment with JTV-519 [10]. Therefore, we tested whether JTV-519 would prevent the delay in calcium reuptake and the increased SERCA activity observed in skeletal muscle fibers after FK506-treatment. 

Indeed, we found that pretreatment of muscle fibers with 1 μM JTV-519 prevented the effects of FK506-treatment after burst activation (Figure 2), suggesting that JTV-519 stabilizes the receptor and thereby prevents RyR1 calcium leakage. This stabilizing effect was visible as a significantly decreased calcium profile AUC after burst activation (17 % compared to FK506-treated fibers) and also a significant decrease in OCR (38 % compared to FK506-treated fibers). 

Some inhibitory effects of JTV-519 on SERCA were described in rabbit ventricular myocytes, but are unknown for mouse skeletal muscle [27]. However, if the effects of JTV-519 on calcium transients were due to SERCA inhibition, OCR would also be reduced by JTV‑519 treatment alone [28]. As this is not the case, we exclude an inhibitory effect of JTV-519 on SERCA activity at a concentration of 1μM. 

At the highest JTV-519 dose tested (10 μM) no calcium transients could be detected (data not shown), indicating that JTV-519 has some off-target effects. JTV-519 has been described as a non-specific blocker of sodium (IC_50_ = 1.2 μM), potassium (IC_50_ = 5 μM) and calcium (IC_50_ = 3 μM) channels in guinea pig ventricular myocytes [29]. 

The exact mode of action of JTV-519 on RyR is still unclear. Most reports focus on the effect of JTV-519 on the cardiac RyR2. It is assumed that the compound stabilizes the closed state of RyR2 by increasing its affinity for calstabin2 and thereby preventing the calcium leak that triggers arrhythmias [10]. Other studies, however, show that calstabin2 is not required for inhibition of the calcium leak by JTV-519 [30]. A recent study suggests that RyR1 calcium leakage as a result of calstabin1 depletion, can be reversed by a number of drugs, including JTV-519, that act as strong electron donors and thereby replace the function of calstabin1 [12].

In summary, we could show that FK506-induced RyR1 receptor leakiness leads to a delay in cytosolic calcium removal after burst activation and an increase of cellular energy consumption. Pretreatment of muscle fibers with 1 μM JTV-519 can partly protect muscle fibers from these effects, presumably by stabilizing the closed state of the receptor.
